# Load Carrying Walking Test for the Special Operation Forces of the Army of the Czech Republic

**DOI:** 10.1093/milmed/usad387

**Published:** 2023-09-30

**Authors:** Martin Bugala, Martina Bernaciková, Ivan Struhár

**Affiliations:** Department of Physical Education and Social Sciences, Faculty of Sports Studies Masaryk University, Brno 625 00, Czech Republic; Department of Physical Activities and Health Sciences, Faculty of Sports Studies Masaryk University, Brno 625 00, Czech Republic; Department of Physical Activities and Health Sciences, Faculty of Sports Studies Masaryk University, Brno 625 00, Czech Republic

## Abstract

**Introduction:**

The subject of this study was the creation of a new type of laboratory walking stress test for the Special Forces of the Army of the Czech Republic. This study developed a test model that has been validated in practice and that reflects the fact that the performance of endurance without and with a load varies considerably. Especially, if we focus on operators, as their activities are always performed with loads/full gear (equipment, weapons, equipment, etc.).

**Materials and Methods:**

24 men/operators from the Special Forces of the Army of the Czech Republic volunteered for this study. The maximal exercise test/spiroergometry was designed to include performance at a load of 55 kg/121 lb, a constant speed of 5.3 km/h, and an increase in incline angle of 1 degree after each elapsed minute. The test was performed on a treadmill under standard time, location, and temperature conditions. During the test, the following values were recorded: VO2 = oxygen consumption indicating the intensity of the exercise was monitored, VO2/kg = oxygen consumption converted to body weight, VO2/HR = pulse oxygen (the amount of oxygen converted in one heartbeat), HR = heart rate, VE = ventilation, volume of air exhaled in 1 min, breathe frequency (BF) = respiratory rate in 1 min.

**Results:**

Out of the total number of 24 respondents, the study found these mean values of variables. The variable mapping the oxygen consumption, which indicates the intensity of VO2 loading, was 3.8, with the lowest value being 3.2 and the highest being 4.5. After converting oxygen consumption to bodyweight, VO2/kg was 46, the lowest value of 38.8, and the highest 53.0 were measured for this variable. Pulse oxygen, i.e., the amount of oxygen calculated per heart contraction VO2/HR, was 20.5, the lowest value 16.0, and the highest 26.0. The average HR heart rate was 183.5, with the lowest value being recorded at 164 and the highest value is 205. Ventilation, i.e., the amount of exhaled air per minute in VE, was measured at 144.9, the lowest value was 114.7, and the highest was 176.6. The BF in 1 min was 58.5, the lowest value 35, and the highest 70. The mean time load was 10:20 min, the shortest test length was 7:25 min, and the longest was 13:23 min. These values correspond to the degree of inclination of the ascent, i.e., the mean value was 10 degrees, the smallest achieved slope was 7 degrees, and the largest 13 degrees.

**Conclusion:**

The designed weighted walking test proved to be fully functional and effective in measurement. The further established protocol corresponds to the requirements of the current needs of the Special Forces of the Army of the Czech Republic. Last but not least, the walking stress test is applied for the external and internal selection and screening of operators. Data obtained from testing were used to develop deployment requirements for patrol/nuclear combat missions.

## INTRODUCTION

Combat performance is the result of general and special training. We understand it as a manifestation of physical and mental abilities or as a manifestation of specialized abilities of a soldier in a conscious activity which is focused on solving combat tasks on the battlefield. Based on these facts, it is clear that combat performance fundamentally affects physical and mental fitness.^1--3^ To reveal the physical fitness of the Army of the Czech Republic elite group from the point of view of exercise physiology, it is necessary to apply sophisticated laboratory testing. Such testing reveals a functional characteristic and determines the starting position for setting the limits of elite soldiers when performing missions in harsh climatic conditions under load.^4,5^ Physiological and anthropometric testing of elite soldiers will also make it possible to identify specific performance indicators that can be used during selection procedures or screenings and to define ideal values for improving aerobic and anaerobic capacity, strength or body fat.^6--9^

The selection of effective candidates itself is one of the most difficult tasks. Therefore, a new load carrying walking test was created in order to meet the needs of special forces in human resources selection. Thanks to the new test, it is possible to reveal in the selection procedure the possible potential for coping with the burden that awaits the candidate in the next selection tasks. Alternatively, the test can also be used to select combat operators for planned operations aimed at patrolling/core tasks of the mission.

As mentioned above, this study developed a test model that has been validated in practice and that reflects the fact that the performance of endurance without and with a load varies considerably. Especially if we focus on operators, as their activities are always performed with loads/full gear (equipment, weapons, equipment, etc.), it is also necessary to mention that those who have a high score, for example, in the Cooper test, may not be able to do aerobic activity under load. Therefore, the study focused on creating a laboratory test model which would make it possible to point out the ability of managing endurance performance under load.

The creation of the load carrying walking test itself was preceded by a thorough search of the literature dealing with physiological testing and performing physical exercise under load, the distribution of the weight of the load and the determination of walking speed and inclination. The weight of the load, speed, and inclination are fundamental quantities that affect the soldier’s performance under load.^10^ Among the facts taken into consideration there are also findings referring to the load on the musculoskeletal system in connection to the weight of the load carried and the speed of walking, i.e., findings related to determining the appropriate load for the functionality of the musculoskeletal system.^11,12^ Last but not least, the study dealt with the lower limbs angular changes which have a vast impact on the health of the musculoskeletal system.^13^

Based on the entry requirements of the Special Forces Command of the Army of the Czech Republic and the knowledge gained from the literature search, a draft of a walking test was created in order to determine in a relatively short time the level of physical condition of the Special Forces operators. The study aims to create a new load carrying walking laboratory test for the operators of the Special Unit of the Czech Army.

## MATERIALS AND METHODS

Twenty-four male participants volunteered and agreed to participate in the presented study. The average age of the participants was 32. The youngest participant was 21 and the oldest one was 46. The standard deviation of the age variable was 6.879. The average body height was 176.5 cm/69.4 inch and the average body weight was 83.6 kg/184.3 lb. The average values of the InBody were as follows: body fat mass was 11.89, fat-free mass was 72.02, skeletal muscle mass was 41.22, body mass index was 26.38, and breathe frequency (BF) was 14.03%.

All participants provided a written informed consent and they were informed of their right to withdraw at any time. The pilot study was aimed at operators of the special forces of the Army of the Czech Republic. No women signed up for the pilot study.

The study was created in cooperation with Masaryk University, the Faculty of Sports Studies, and the Army of the Czech Republic. The selection of the research sample was based on voluntariness. Twenty-four male subjects were chosen from the Czech Army Special forces to participate in this study. The study was focused on creating a new laboratory test to map the maximum oxygen consumption when carrying the load of an operator. The maximum load test was adjusted to incorporate power under load to ensure timely distribution and possible monitoring of physiological functions. As a first step, it was important to search the kind of equipment and average weight which is usually carried by operators. Instructors of Special Forces chose the appropriate weight, gear, equipment, weapons, communication equipment, ammunition, and field equipment. After having described the result, a load of 55 kg/121 lb was chosen. Furthermore, a constant walking speed of 5.3 km/h (3.3 MPH) was set. This is the usual walking speed of healthy adults on level ground. However, because of the fact that the research group was composed of elite operators, an increase in inclination of 1% was included in the test after each minute.

### VO2max Test

The maximum load test was completed on a motorized treadmill (Lode Katana, Groningen, The Netherlands). The gas analysis system (Cortex Metalyzer, Lepzig, Germany) was calibrated before each test in accordance with the manufacturer’s instructions. All VO_2_max test took place in the same temperature-regulated laboratory (22.0 ± 0.5 °C/71.6 ± 32.9°F) with the relative humidity of ±35%). Before the experimental testing protocol, all participants were instructed to be hydrated ad libitum and to refrain from strenuous exercise and from consuming alcohol or caffeine for 24 h.

Following a period of self-selected warm-up, each participant completed an incremental step-test to volitional exhaustion in which oxygen consumption (Cortex Metalyzer, Lepzig, Germany) and heart rate (Polar Heart Rate Chest H10+) were recorded for the time of testing protocol. For the purpose of this study, the plateau in V̇O_2_ stands out as the main criterion of test validity of oxygen between two consecutive stages.

The speed of the treadmill remained constant at 5.3 km/h (3.3 MPH) while the gradient increased at a rate of 1% every 60 s from the initial starting gradient of 0%. The treadmill test was terminated when the participant either reached volitional exhaustion or consistently showed an inability to walk with 55 kg/121 lb bag. At the end of each test, the rate of perceived exertion was determined on a Borg Scale (6–20) Each participant was verbally encouraged and motivated by a researcher to reach the maximum exercise capacity.

**FIGURE 1.**
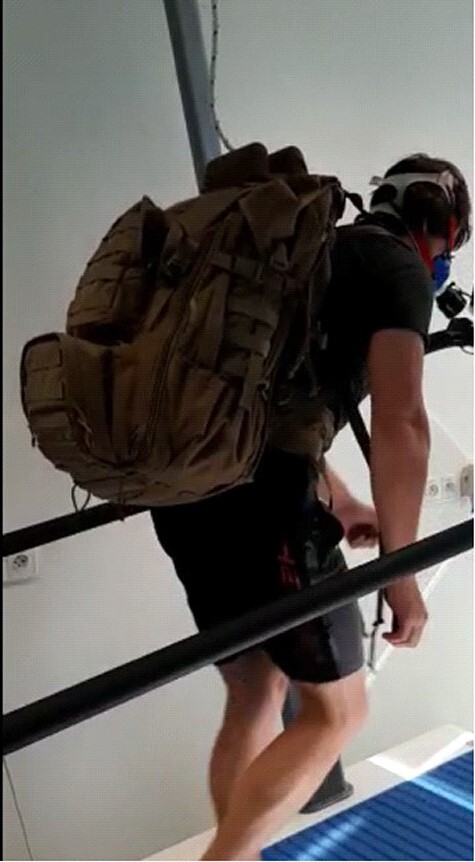
Load walking test.

After putting on a backpack weighing 55 kg/121 lb, the measured subject was moved to a treadmill and anchored in case of a fall (Fig. 1). The termination of the subject by will was determined. The subject was allowed to finish the testing whenever they required.

## RESULTS

The obtained results are demonstrated using two tables where Table I shows the values of each respondent who passed the walking test. These physiological parameters of individual probands indicate that each of them passed a set gait test and their data were processed. It also shows the background of the overall rating in Table II. The obtained values of all respondents after the implemented load walking test are showed in Table II. The variable’s average, median, minimum, maximum values, and standard deviation were used for individual variables. These basic statistical indicators are performed by the overall description of the research group after the load walking test.

**TABLE I.** Lists the Physiological Load Parameters and Test Time for All Participating Test Soldiers

ID	VO2	VO2/kg	VO2/HR	HR	VE	BF	Time	Tilt %	Height (cm)	Height (inch)	Weight (kg)	Weight (lb)	Year of birth
001	4.01	48	23	177	155.2	58	0:08:19	8.00	170	66.9	83.6	184.3	1982
002	3.80	46	22	171	158.6	70	0:10:21	10.00	178	70.0	81.9	180.5	1976
003	3.57	42	18	197	132.6	48	0:09:31	9.00	187	73.6	85.7	188.9	1993
004	3.88	53	21	187	123.4	47	0:10:38	10.00	178	70.0	73.1	161.1	1999
005	3.93	49	21	188	126.3	50	0:11:19	11.00	177	69.6	81.1	178.7	2000
006	3.43	46	18	186	131.2	60	0:10:20	10.00	170	66.9	74.2	163.5	1999
007	4.29	44	23	183	163.8	56	0:10:21	10.00	186	73.2	96.8	213.4	1996
008	3.37	46	19	180	123.2	51	0:10:17	10.00	182	71.6	73.2	161.3	1997
009	4.47	50	25	181	176.2	64	0:12:25	12.00	184	72.4	88.8	195.7	1991
010	4.35	52	26	171	160.2	62	0:10:33	10.00	171	67.3	83.8	184.7	1980
011	4.32	46	24	183	122.6	35	0:10:51	10.00	193	75.9	93.4	205.9	1989
012	3.66	46	20	184	132.4	60	0:10:20	10.00	172	67.7	80.0	176,3	1993
013	3.97	49	22	184	149.6	42	0:10:35	10.00	175	68.8	80.7	177.9	1988
014	3.92	46	20	200	159.7	54	0:12:25	12.00	178	70.0	85.2	187.8	1994
015	3.86	46	24	164	172.4	64	0:08:20	8.00	173	68.1	83.6	184.3	1975
016	3.20	40	16	194	142.1	69	0:07:31	7.00	173	68.1	79.0	174.1	1990
017	3.44	39	20	174	114.7	59	0:08:17	8.00	176	69.2	88.9	195.9	1992
018	4.23	47	22	190	159.0	57	0:12:20	12.00	176	69.2	90.4	199.2	1987
019	3.78	50	21	176	141.4	65	0:08:38	8.00	170	66.9	75.7	166.8	1982
020	3.61	39	20	178	147.6	54	0:10:15	10.00	186	73.2	93.3	205.6	1986
021	3.99	43	20	204	133.3	47	0:13:23	13.00	193	75.9	91.9	202.6	1994
022	3.31	41	18	179	133.6	65	0:10:20	10.00	176	69.2	79.9	176.1	1988
023	3.32	38	18	187	152.5	62	0:07:25	7.00	175	68.8	88.2	194.4	1991
024	3.77	46	20	189	148.3	62	0:10:20	10.00	182	71.6	81.8	180.3	1992

**Abbreviations**: BF = breathe frequency in 1 min [IS1];time = time of the test; HR = heart rate; ID = identification of the respondent; Tilt = treadmill tilt angle.; VE = ventilation, amount of air exhaled in 1 min; VO2 = oxygen consumption, which indicates the intensity of the load; VO2/HR = pulse oxygen (amount of oxygen converted to one heartbeat); VO2/kg = converted oxygen consumption to body weight.

**TABLE II.** General Description of Physiological Values of the Walking Test

Variable	Valid N	Mean	Median	Mini	Maxi	SD
VO2	24	3.8	3.8	3.2	4.5	0.4
VO2/kg	24	45.5	46.0	38.0	53.0	4.1
VO2/HR	24	20.9	20.5	16.0	26.0	2.5
HR	24	183.6	183.5	164.0	204.0	9.5
VE	24	144.2	144.9	114.7	176.2	16.9
BF	24	56.7	58.5	35.0	70.0	8.7
Time	24	10:13	10:20	7:25	13:23	1:33
Tilt %	24	9.8	10.0	7.0	13.0	1.6
Height (cm/inch)	24	178.4/70.2	176.5/69.4	170.0/66.9	193.0/75.9	6.9
Weight (kg/lb)	24	83.9/184.9	83.6/184.3	73.1/161.1	96.8/213.4	6.6

**Abbreviations**: BF = breathe frequency in 1 min [IS1]; height = the height of the proband]; HR = heart rate]; ID = identification of the respondent; tilt = treadmill tilt angle; time = time of the test; VE = ventilation, amount of air exhaled in 1 min; VO2 = oxygen consumption, which indicates the intensity of the load; VO2/HR = pulse oxygen (amount of oxygen converted to one heartbeat); VO2/kg = converted oxygen consumption to body weight; weight = weight of the proband.

Out of the total number of 24 respondents, the study found these mean values of variables. The variable mapping the oxygen consumption, which indicates the intensity of VO2 loading, was 3.8, with the lowest value being 3.2 and the highest being 4.5. After converting oxygen consumption to bodyweight, VO2/kg was 46, the lowest value of 38.8, and the highest 53.0 were measured for this variable. Pulse oxygen, i.e., the amount of oxygen calculated per heart contraction VO2/HR, was 20.5, the lowest value 16.0, and the highest 26.0. The average HR heart rate was 183.5, with the lowest value being recorded at 164 and the highest value is 205. Ventilation, i.e., the amount of exhaled air per minute in VE, was measured at 144.9, the lowest value was 114.7, and the highest was 176.6. The BF in 1 min BF was 58.5, the lowest value 35, and the highest value 70. The mean time load was 10:20 min, the shortest test length was 7:25 min, and the longest 13:23 min. These values correspond to the degree of inclination of the ascent, i.e., the mean value was 10 degrees, the smallest achieved slope was 7 degrees, and the largest 13 degrees. It should be noted that a constant speed of 5.3 km/h (3.3 MPH) was still maintained. The output values obtained by the test are defined as the highest value of the moving average in 15 s (typically before the end of the test). The last two variables in Table II demonstrate the mean values of height and weight. The average height was calculated at 176.5 cm/69.4 inch, and the smallest respondent measured 170 cm/66.9 inch and the highest 193 cm/75.9 inch. The average weight was 83.6 kg/184.3 lb, the lightest respondent weighed 73.1 kg/161.1 lb, and the heaviest was 96.8 kg/213.4 lb.

## DISCUSSION

Laboratory testing of fitness skills is a sophisticated way of determining the current state of the athlete in our case, the operator. This statement is by no means new or revolutionary. However, what is “revolutionary” is to create effective tests that in a relatively short time reveal the ability or fitness to perform the profession.

No-load monitoring studies in the military environment are often an indicator of aerobic fitness.^14,15^ This knowledge is suitable for sports, for a physiological description of operators, or for correlation of field tests with laboratory.^16^ However, the operators’ activity is performed with a load, i.e., the overall problem arises when a load in the form of equipment is added to the cyclic movement, such as walking or running. That is way it was necessary to create a test that checks the overall fitness readiness of the operator. Of course we are aware that the so far studies have always worked with different types of exercise load and addressed health and metabolic aspects in different types of exercise, speed, and gender.^16--18^

However, none of the tests has been incorporated into the selection of operators and none of them has worked with a slope that fundamentally affects performance. It is also necessary to mention that a continuous load at a certain constant speed creates a certain movement stereotype. Thus, walking speed and inclination in fixed values lead to a linear increase or decrease in performance over the time.^19^ However, reaching the peak of the test would be time-consuming. This fact was taken into account and thanks to the increasing elevation of the treadmill there is a constant increase in muscle work and in oxygen consumption and the mentioned peak of the test is achieved. For this reason, we believe the test is effective. However, it is necessary to realize that it is still a laboratory test, so it is possible to work with anthropometric facts, biomechanics, and exercise physiology.

Nevertheless, the real conditions are always different. It means that different variables enter into the performance itself and this fact fundamentally influences the operator’s performance. It is, therefore, to be taken into account that, for example, weather conditions, topographic relief, surface conditions and temperature changes largely affect the overall production of the operator.^20^ Furthermore, the operator is also required to fulfill concrete combat tasks and various combative (self-defense) and other combat technical means (positions, postures, transitions, movements of legs and arms, turns, moving, carrying, and lowering a live load)^21,22^ are supposed to be carried out as well.

Therefore, it reveals to be very appropriate to adjust the test in order to have the elevation and thermal load taken into consideration. That would result in simulating better the real conditions while testing is carried out, in particular, conditions typical for mountainous terrain with high temperatures.^4^ We would achieve a kind of testing that would be more complex and other aspects affecting the operator’s performance, such as hypohydration, could be examined as well.^23^

Unfortunately, no women signed up for the pilot study. We believe that the participation of women would greatly enrich the testing. However, a discussion could be opened about whether the weight of 55 kg/ 121 lb could possibly be a partial limitation for female operators. There is no doubt that including women in this testing could be a great challenge and in the future we would like to apply this test to the entire special forces.

## CONCLUSIONS

Continuous observing and testing of operators is a way of monitoring their performance, state of health and physiological functions under load. For this reason, there is a great necessity of continuous updating and creating new test batteries and methods which can provide some exact data for drawing up analyses and making predictions about the future development, professional orientation, or injury risks in single operators.^24--26^ The purpose of the study was not to compare data from various other load tests, as the measurement processes and structures are completely different. The study wanted to point out that the operators must have at their disposal some more realistic fitness tests because the classic laboratory or field tests cover only the general fitness of common population, and not the professional fitness of the army operators.

As a result of this study there is a proposal of a new load carrying walking test created particularly for the army operators. The test itself is based on the concrete needs of the Command of the Special Forces of the Czech Army whose best responsibility it is to recognize in full the real complexity of the work of operators. The present test model has been able to satisfy the needs and requirements of the aforementioned Special Forces Command. The pedestrian load carrying walking test has already been applied for external and internal selection procedures and for screening of operators. The data obtained from testing was used to create requirements for deployment on combat missions aimed at patrolling/core tasks.

## Data Availability

The data that support the findings of this study are available upon request from the corresponding author.
